# Serum metabolomics analysis reveals metabolite profile and key biomarkers of idiopathic membranous nephropathy

**DOI:** 10.7717/peerj.15167

**Published:** 2023-04-06

**Authors:** Mingjun Ye, Donge Tang, Weilong Li, Chiyu Ma, Zhipeng Zeng, Shengyou Liao, Zhuoheng Song, Yu Meng, Fanna Liu, Shaodong Luan, Lianghong Yin, Yong Dai

**Affiliations:** 1 Clinical Medical Research Center, The Second Clinical Medical College of Jinan University, Shenzhen People’s Hospital, Shenzhen, Guangdong, China; 2 Institute of Nephrology and Blood Purification, the First Affiliated Hospital of Jinan University, Jinan University, Guangzhou, China; 3 Department of Nephrology, Shenzhen Longhua District Central Hospital, Shenzhen, China

**Keywords:** Idiopathic membranous nephropathy, Biomarker analysis, Metabolomics, Dehydroepiandrosterone sulfate, Gut microbes

## Abstract

**Background:**

Idiopathic membranous nephropathy (IMN) is an organ-specific autoimmune disease with multiple and complex pathogenic mechanisms. Currently, renal biopsy is considered the gold standard for diagnosing membranous nephropathy. However, there were limitations to the renal puncture biopsy, such as the relatively high cost, longer time consuming, and the risk of invasive procedures. We investigated the profile of serum metabolites in IMN patients based on the UHPLC-QE-MS metabolomics technique for exploring the potential disease biomarkers and clinical implementation.

**Methods:**

In our research, we collected serum samples from healthy control (*n* = 15) and IMN patients (*n* = 25) to perform metabolomics analysis based on the UHPLC-QE-MS technique.

**Result:**

We identified 215 differentially expressed metabolites (DEMs) between the IMN and healthy control (HC) groups. Furthermore, these DEMs were significantly identified in histidine metabolism, arginine and proline metabolism, pyrimidine metabolism, purine metabolism, and steroid hormone biosynthesis. Several key DEMs were significantly correlated with the level of clinical parameters, such as serum albumin, IgG, UTP, and cholesterol. Among them, dehydroepiandrosterone sulfate (DHEAS) was considered the reliable diagnostic biomarker in the IMN group. There was an increased abundance of actinobacteria, phylum proteobacteria, and class gammaproteobacterial in IMN patients for host-microbiome origin analysis.

**Conclusion:**

Our study revealed the profiles of DEMs from the IMN and HC groups. The result demonstrated that there were disorders of amino acids, nucleotides, and steroids hormones metabolism in IMN patients. The down-regulation of DHEAS may be associated with the imbalance of the immune environment in IMN patients. In host-microbiome origin analysis, the gut microbiota and metabolite disturbances were present in IMN patients.

## Introduction

As a major cause of nephrotic syndrome (NS), membranous nephropathy (MN) accounts for approximately 20–30% of NS in adults, and the pathological feature is diffuse immune complex deposition under the epithelial cells of the glomerular basement membrane (GBM) with thickening of the basement membrane (Cattran & Brenchley, 2017; Ronco & Debiec, 2015). MN can be mainly divided into idiopathic membranous nephropathy (IMN) and secondary membranous nephropathy (SMN), and secondary etiologies include autoimmune diseases, infections, drugs, and tumors (Nature Reviews Disease Primers, 2021; Lai et al., 2015). Patients with membranous nephropathy mainly present with varying degrees of proteinuria, and the level of urinary protein is correlated with the renal prognosis (Chen et al., 2019a). IMN patients with persistent high-grade proteinuria will gradually progress to end-stage renal disease (ESRD) (Thompson et al., 2015). The pathogenesis of IMN is the result of comprehensive factors. Numerous studies have demonstrated multiple and complex pathogenic mechanisms of IMN, including IgG4 Abs against the M-type phospholipase A2 receptor (PLA2R), thrombospondin type 1 domain-containing 7A (THSD7A), neutral endopeptidase (NEP), and other mechanisms not yet identified (Akiyama, Imai & Maruyama, 2019; Cattran & Brenchley, 2017; Couser, 2017; Kerjaschki, 2004). IMN is currently diagnosed with renal biopsy as the gold standard, but the repetitive clinical implementation of renal biopsy is limited to the relatively high cost, longer time consumption, and the risk of invasive procedures (Bobart et al., 2019). Monitoring and efficacy of IMN still rely heavily on clinical parameters of urine protein and renal function, which lack specificity (Logt et al., 2021; van den Brand, Hofstra & Wetzels, 2011). Therefore, exploring sensitive, specific, and non-invasive biomarkers have great clinical significance for diagnosing and treating membranous nephropathy.

Metabolomics is dedicated to the analysis of small molecule metabolites in living organisms, providing new insights for biological and clinical research (Kalim & Rhee, 2017). It can be combined with multi-omics analysis and is expected to be a powerful tool for clinical implementation (Johnson, Ivanisevic & Siuzdak, 2016; Rinschen et al., 2019). Therefore, we explored the diversity of serum metabolites in IMN patients based on the non-targeted metabolomics analysis, which provided the basis for further exploring the potential diagnosis biomarkers, disease monitoring, and efficacy determination of idiopathic membranous nephropathy.

## Materials and Methods

### Patients

We recruited 25 patients diagnosed with idiopathic membranous nephropathy from the First Affiliated Hospital of Jinan University. The ages of all IMN patients ranged from 40 to 73 years. Likewise, our study also recruited 15 age-matched people for healthy controls. All IMN patients underwent renal biopsy to confirm the diagnosis. The inclusion and exclusion criteria of our study were shown in the Supplemental Materials. Our research was approved by the Helsinki Declaration of ethical principles for medical research about human subjects and the ethics committee of the First Affiliated Hospital of Jinan University (KY-2021-018). We collected the serum samples after obtaining the written informed consent of all participants.

### Metabolomics analysis

#### Metabolites extraction

In our experiment, 400 μL of solvent (acetonitrile:methanol = 1:1) was added to 100 μL of serum samples and vortexed for 30 s. The samples were sonicated for 10 min in an ice-water bath, incubated for an hour at −40 °C, and centrifuged for 15 min at 4 °C at 12,000 rpm. A total of 120 μL of supernatant was transferred to a sample vial in preparation for further LC-MS/MS analysis. The quality control (QC) sample was prepared by mixing an equal aliquot of the supernatants from all samples.

#### LC-MS/MS analysis

We used a UHPLC system (Vanquish; Thermo Fisher Scientific, Waltham, MA, USA) with a UPLC BEH Amide column (2.1 mm × 100 mm, 1.7 μm) combined with Q Exactive HFX mass spectrometer (Orbitrap MS; Thermo, Waltham, MA, USA) to perform LC-MS/MS analysis. The mobile phase A was formed with 25 mmol/L ammonium acetate and 25 mmol/L ammonia hydroxide in water (pH = 9.75), and the mobile phase B was acetonitrile. The temperature of the auto-sampler was 4 °C, and the injection volume was 3 μL. We acquired MS/MS spectra using the QE HFX mass spectrometer on the information-dependent acquisition (IDA) mode under the control of the acquisition software (Xcalibur; Thermo, Waltham, MA, USA). The detailed steps and parameter settings of LC-MS/MS analysis and data preprocessing and annotation were described in the Supplemental Materials.

#### Bioinformatics methods

The dataset containing the sample name, normalized peak area, peak number, retention times, and m/z pairs was imported to the SIMCA16.0.2 software package for multivariate analysis, including principal component analysis (PCA) and orthogonal projections to latent structures-discriminate analysis (OPLS-DA). Scaling and logarithmic transformation of data to minimize the effects of noise and high variance of the variables. And the value of variable importance in the projection (VIP) of the first principal component in OPLS-DA analysis was obtained. *P*-values were derived from a two-tailed student’s t-test on normalized peak areas of DEMs between the two groups. Fold change was the ratio of the relative quantification of DEMs between the IMN group and HC group, derived from the univariate analysis. The criterion of differentially expressed metabolites was defined as the *p*-value < 0.05 and VIP > 1. Furthermore, we used R version 4.0.4 to create the visual graphs as described below. The permutation test of the OPLS-DA model was applied by the R package “ggplot2”. The process quality control with good stability was shown in Figs. S2–S5. Volcano plot and violin plots were performed with the R package “ggplot2”, and the radar chart was performed with R package “fmsb”. The pathway enrichment, statistical analysis, and biomarker analysis were visualized with MetaboAnalyst 5.0. Moreover, we used MetaboAnalyst 5.0 to create the heat map and receiver operating characteristic (ROC) curve analyses using the biomarker analysis module. Metorigin 2.0 was conducted on host-microbiome origin analysis of differentially expressed metabolites. The detailed methods can be refered to the Supplemental Materials.

### Statistical analysis

Our study performed the statistical analysis by GraphPad Prism 9.0 software, using two-tailed Student’s *t*-tests for measurement data. A *p*-value less than 0.05 indicates a statistically significant difference. We applied Spearman correlation analysis to analyze correlations between DEMs and clinical parameters. The correlation analysis and correlation network were visualized with the OmicStudio tools.

## Result

### Basic characteristics of participants

To investigate the effects of serum metabolites in IMN patients, our study performed metabolomic analysis for 40 serum samples based on LC-MS/MS system from the IMN group and HC group. There were no significant differences in the general information, such as age, sex, and BMI. We summarized specimen information in Table 1. Statistical analysis of clinical specimen information revealed that some clinical indicators were not statistically different, such as alanine transaminase (ALT), aspartate transaminase (AST), creatinine, high-density lipoprotein (HDL), and triglyceride (TG) (*p* > 0.05). There were significant differences in serum albumin, cholesterol, low-density, lipoprotein (LDL), 24-h proteinuria, uric acid (UA), blood urea nitrogen (BUN), IgG, and Cys-C between the two groups (*p* < 0.05). For IMN patients, levels of serum albumin and lgG were significantly decreased; however, levels of cholesterol, LDL, BUN, 24-h proteinuria, and UA were significantly increased. This was consistent with the clinical presentation of membranous nephropathy, and IMN patients often present with profuse proteinuria, hypoproteinemia, oedema, and hyperlipidemia.

**Table 1:** Clinical characteristics of healthy controls and idiopathic membranous nephropathy patients.

Characteristics	Healthy control group (*n* = 15)	IMN group (*n* = 25)
Gender (male/female)	4/11	12/13
Age (years)	53.13 ± 7.32	58.68 ± 10.00
BMI (kg/m^2^)	22.29 ± 1.76	23.64 ± 2.92
SBP (mmHg)	116.20 ± 8.71	125 ± 14.17*
ALT (U/L)	14.80 ± 5.12	22.00 ± 14.06
AST (U/L)	18.20 ± 4.65	19.92 ± 8.20
FBG (mmol/L)	5.49 ± 0.41	5.27 ± 1.30
BUN (mmol/L)	4.54 ± 0.41	7.398 ± 3.14**
Creatinine (umol/L)	68.65 ± 8.75	80.96 ± 27.89
Uric acid (umol/L)	287.20 ± 32.25	414.80 ± 110.60***
Serum albumin (g/L)	40.88 ± 2.65	27.94 ± 7.10****
cholesterol (mmol/L)	4.34 ± 0.47	6.61 ± 2.17***
Triglyceride (mmol/L)	1.51 ± 0.21	2.24 ± 1.44
HDL (mmol/L)	1.22 ± 0.08	1.41 ± 0.37
LDL (mmol/L)	2.57 ± 0.14	3.84 ± 1.52**
UTP (mg/24 h)	NA	3.15 ± 2.86
Cys-C (mg/L)	0.94 ± 0.14	1.44 ± 0.55**
lgG (g/L)	9.68 ± 1.42	7.17 ± 2.58**

**Notes:**

* *p* < 0.05.

** *p* < 0.01.

*** *p* < 0.001.

**** *p* < 0.0001.

BMI, body mass index; SBP, systolic blood pressure; ALT, alanine transaminase; AST, aspartate transaminase; FBG, fasting blood glucose; BUN, blood urea nitrogen; HDL, high-density lipoprotein; LDL, low-density lipoprotein; UTP, 24 h urinary total protein; lgG, Immunoglobulin G.

### Community metabolomics profile

**Figure 1:**
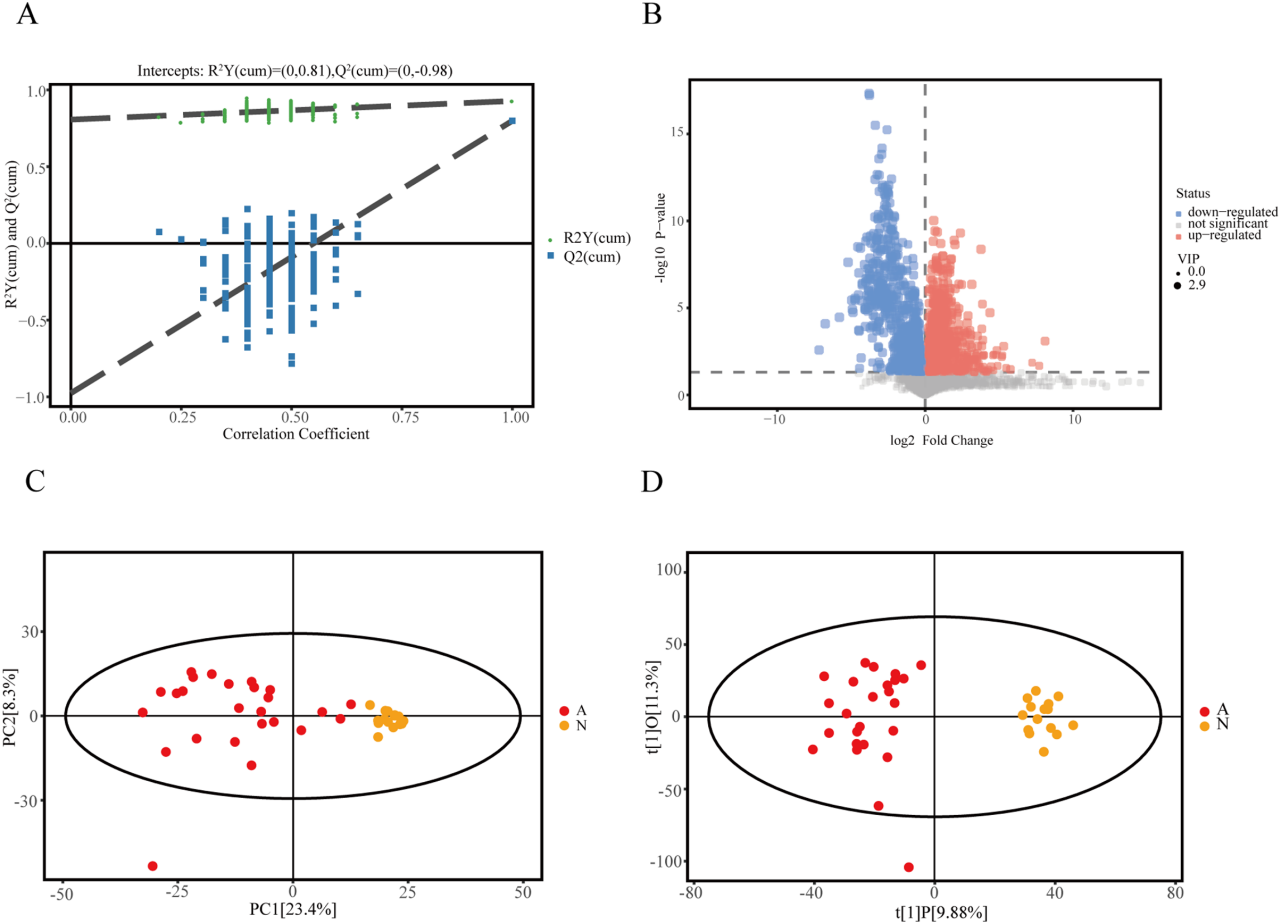
Identification of DEMs in idiopathic membranous nephropathy. (A) Permutation test of OPLS-DA model for the IMN and HC groups. (B) Volcano plot for the IMN and HC groups, each point represents a metabolite with significantly up-regulated DEMs shown in red, significantly down-regulated DEMs shown in blue, and non-significantly expressed metabolites are shown in gray. (C) Score scatter plot of PCA model for the IMN and HC groups. (D) Score scatter plot of OPLS-DA model for IMN and HC groups. OPLS-DA, orthogonal projections to latent structures-discriminate analysis; IMN, idiopathic membranous nephropathy; HC, healthy control; DEMs, differently expressed metabolites; PCA, principal component analysis.

We recruited 25 IMN patients and 15 healthy controls to identify serum metabolites based on the UHPLC-QE-MS technique. Firstly, a series of data management was processed, such as filtering individual peaks to remove noise, filtering on individual peaks, missing value recording, and normalization. The results of the PCA score plot illustrated that the samples were largely within the 95% confidence interval (Fig. 1C). We also analyzed the results using the statistical method of OPLS-DA and permutation test of the OPLS-DA model, which can demonstrate the separation feature between the IMN group and HC group relatively well, with good repeatability and stability and no overfitting (Figs. 1A and 1D). We visualized the screening results for DEMs in a volcano plot (Fig. 1B). As a result, 215 metabolites were defined as DEMs. Moreover, 104 metabolites were identified as up-regulated types (log2(FC) > 0, *p* < 0.05), and 111 DEMs were down-regulated (log2(FC) < 0, *p* < 0.05).

### Identification of metabolites in serum samples

**Figure 2:**
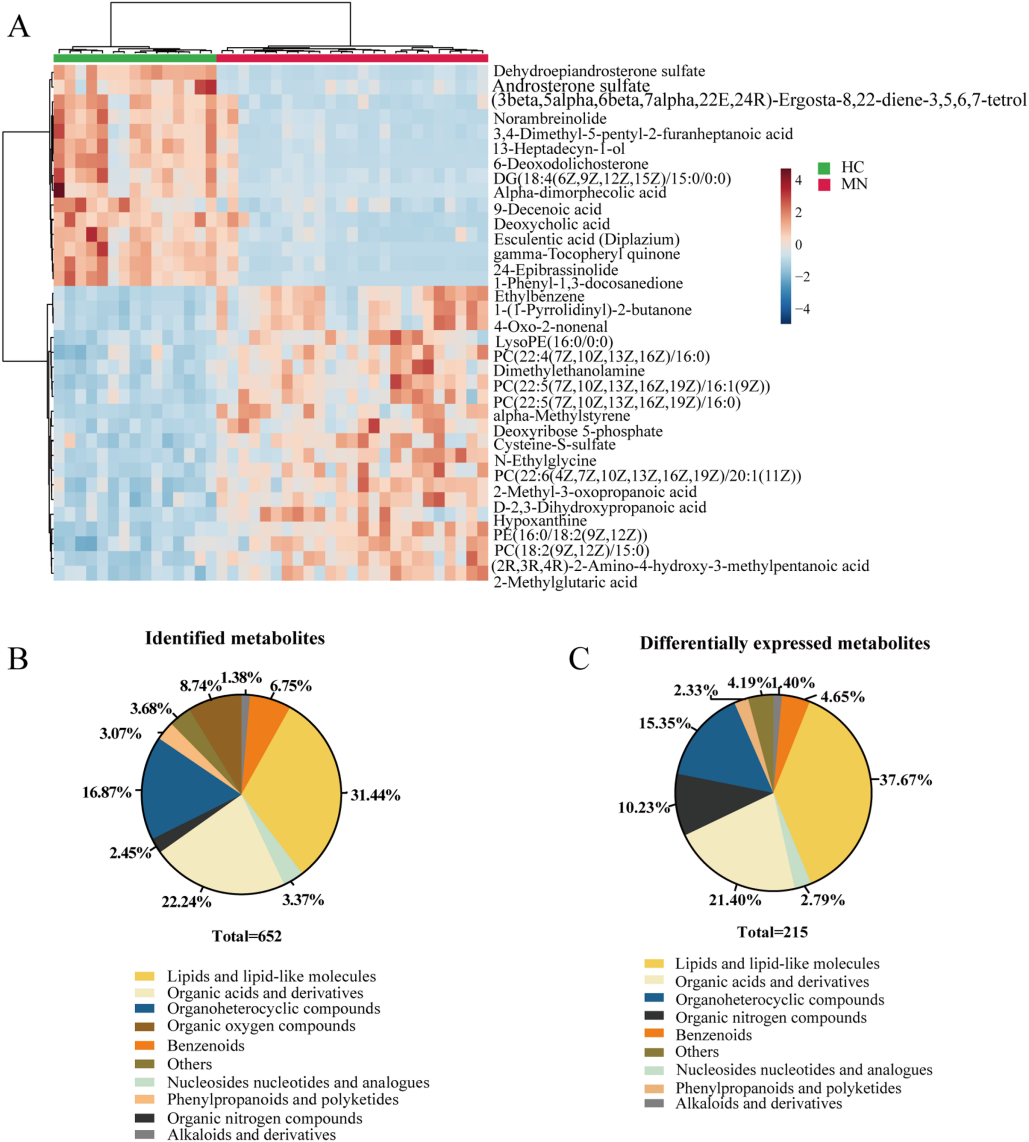
Metabolites profiling in IMN and HC groups. (A) Heatmap of the hierarchical clustering analysis for the top 35 DEMs. The abundance of each metabolite was normalized by Z-score and log2 transformation. (B) The class composition of the whole identified metabolites for the IMN and HC groups. (C) The class composition of the DEMs for the IMN and HC groups.

In our study, we performed the metabolomic analysis in serum metabolites from IMN patients and healthy controls based on the UHPLC-QE-MS technique. As a result, 652 metabolites were successfully identified, and 215 metabolites were significantly altered. The identified metabolites can be categorized as follows: lipids and lipid-like molecules, organoheterocyclic compounds, organic acids and derivatives, benzenoids, organic oxygen compounds, phenylpropanoids and polyketides, and nucleosides nucleotides and analogues. The result showed that lipids and lipid-like molecules accounted for 31.44% of identified metabolites and 37.67% of differentially expressed metabolites. Likewise, organic acids and derivatives for 22.24% of all identified metabolites and 21.40% in DEMs (Figs. 2B and 2C). To some extent, it indicated the effects of lipid metabolic disorders in patients with idiopathic membranous nephropathy.

### Metabolic KEGG pathway enrichment analysis

In addition, we performed statistical analysis for the identified metabolites using the online graphing tool MetaboAnalyst 5.0, and the data were normalized by sum and set auto-scaling. Unsupervised clustering analysis of the membranous nephropathy serum metabolites shows that ethylbenzene, 1-(1-Pyrrolidinyl)-2-butanone, LysoPE (16:0/0:0), dimethylethanolamine, alpha-methylstyrene, deoxyribose 5-phosphate cysteine-S-sulfate, N-ethylglycine, 2-methyl-3-oxopropanoic acid D-2,3-dihydroxypropanoic acid hypoxanthine, and 2-methylglutaric acid were significantly expressed in disease group (Fig. 2A). These metabolites were significantly up-regulated in IMN group. However, dehydroepiandrosterone sulfate, androsterone sulfate, norambreinolide, 3,4-dimethyl-5-pentyl-2-furanheptanoic acid, 13-heptadecyn-1-ol, and 6-deoxodolichosterone were observably clustered in the control group.

**Figure 3:**
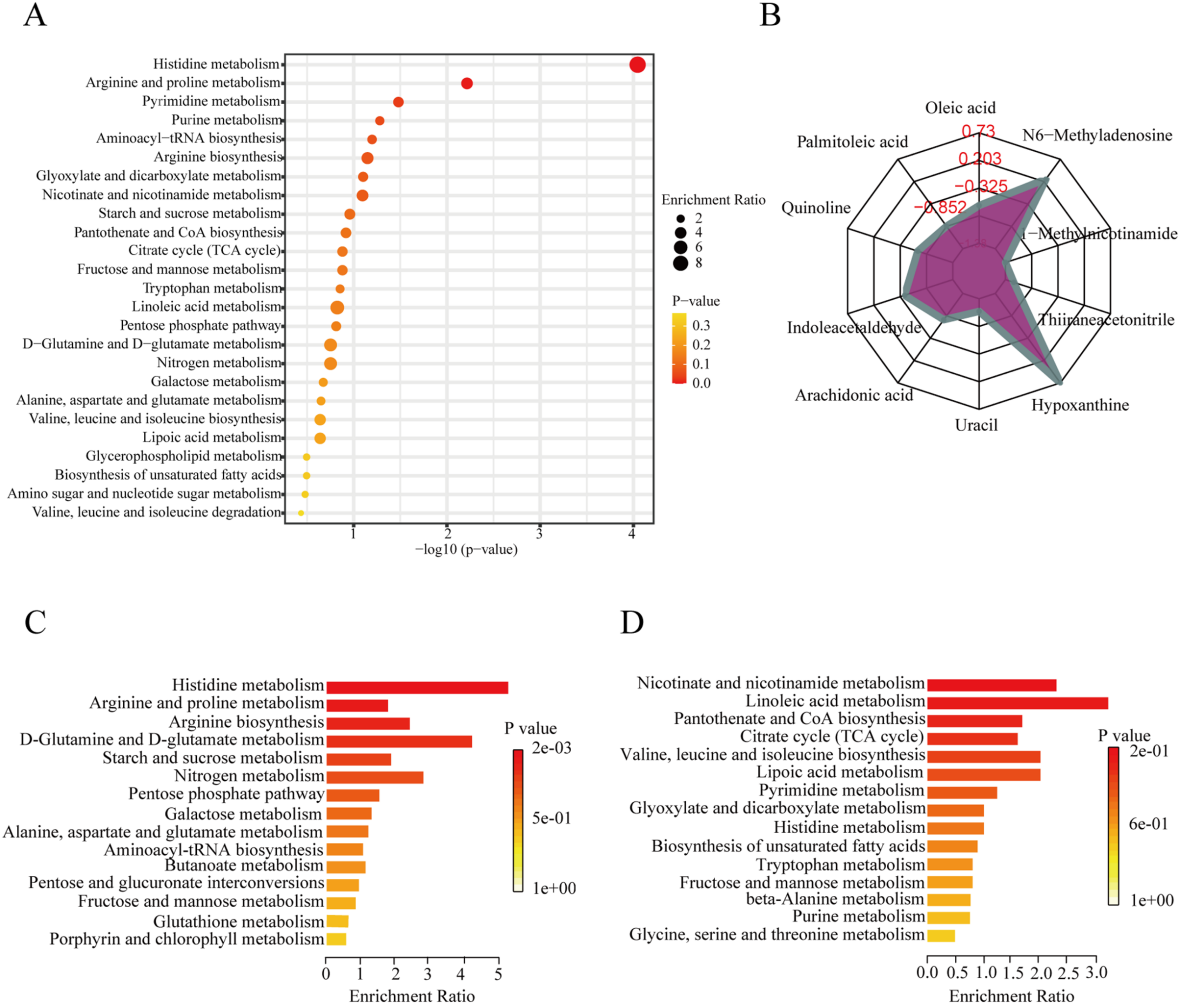
KEGG pathway enrichment analysis of DEMs between IMN and HC groups. (A) The top 25 KEGG pathway enrichment analysis of the 215 DEMs. (B) The numbers in the grid lines represented the log2 fold changes of DEMs for IMN group compared to HC group, and the purple shading consists of a line connecting the log2(FC) for each DEM in the radar chart. (C) The up-regulated KEGG pathway enrichment analysis of DEMs. (D) The down-regulated pathway enrichment analysis of DEMs. KEGG, Kyoto Encyclopedia of Genes and Genomes.

To further explore the role of the DEMs in membranous nephropathy, we conducted KEGG pathway enrichment analysis for the 215 significantly expressed metabolites. We respectively showed the top 25 KEGG pathways, such as histidine metabolism, purine metabolism, arginine and proline metabolism, and aminoacyl-tRNA biosynthesis (Fig. 3A). Compared with the other pathways, the histidine metabolism pathway was listed as the most significant pathway. L-glutamic acid, urocanic acid, L-histidine, imidazole-4-acetaldehyde, 3-methylhistidine, and formiminoglutamic acid were identified in the histidine metabolism pathway. Similarly, the purine metabolism pathway also drew our attention. Hypoxanthine, inosine, guanine, guanosine, and uric acid were enriched in the purine metabolism pathway. The results showed that idiopathic membranous nephropathy patients presented lower levels of imidazole-4-acetaldehyde, guanine, and guanosine compared to the control group. L-glutamic acid, urocanic acid, L-histidine, 3-methylhistidine, formiminoglutamic acid, hypoxanthine, and inosine were identified as up-regulated types in the IMN group. And the corresponding content trend changes of DEMs were displayed in the radar chart. The corresponding metabolites were shown in Fig. 3B, including oleic acid (log2(FC)= −0.66), palmitoleic acid (log2(FC) = −1.38), uracil (log2(FC) = −1.14), hypoxanthine (log2(FC) = 0.73), thiiraneacetonitrile (log2(FC)= − 1.21), 1-methylnicotinamide (log2(FC)= − 1.38), and N6-methyladenosine (log2(FC) = 0.26). The up-regulated DEMs were remarkably enriched in histidine metabolism, arginine biosynthesis, arginine and proline metabolism, D-glutamine and D-glutamate metabolism KEGG pathways in the IMN group (Fig. 3C). Moreover, the down-regulated DEMs were significantly enriched in nicotinate and nicotinamide metabolism, linoleic acid metabolism, pantothenate and CoA biosynthesis, citrate cycle (TCA cycle), lipoic acid metabolism, and biosynthesis of unsaturated fatty acids (Fig. 3D). The results indicated that a series of changes for serum metabolites and metabolic recoding occurred in the blood circulation of IMN patients. Some studies suggested that metabolic changes altered the epigenetic landscape in the disease processes and occurred metabolic reprogramming at multiple levels (Wang & Lei, 2018).

### Diagnostic performance of metabolites

**Figure 4:**
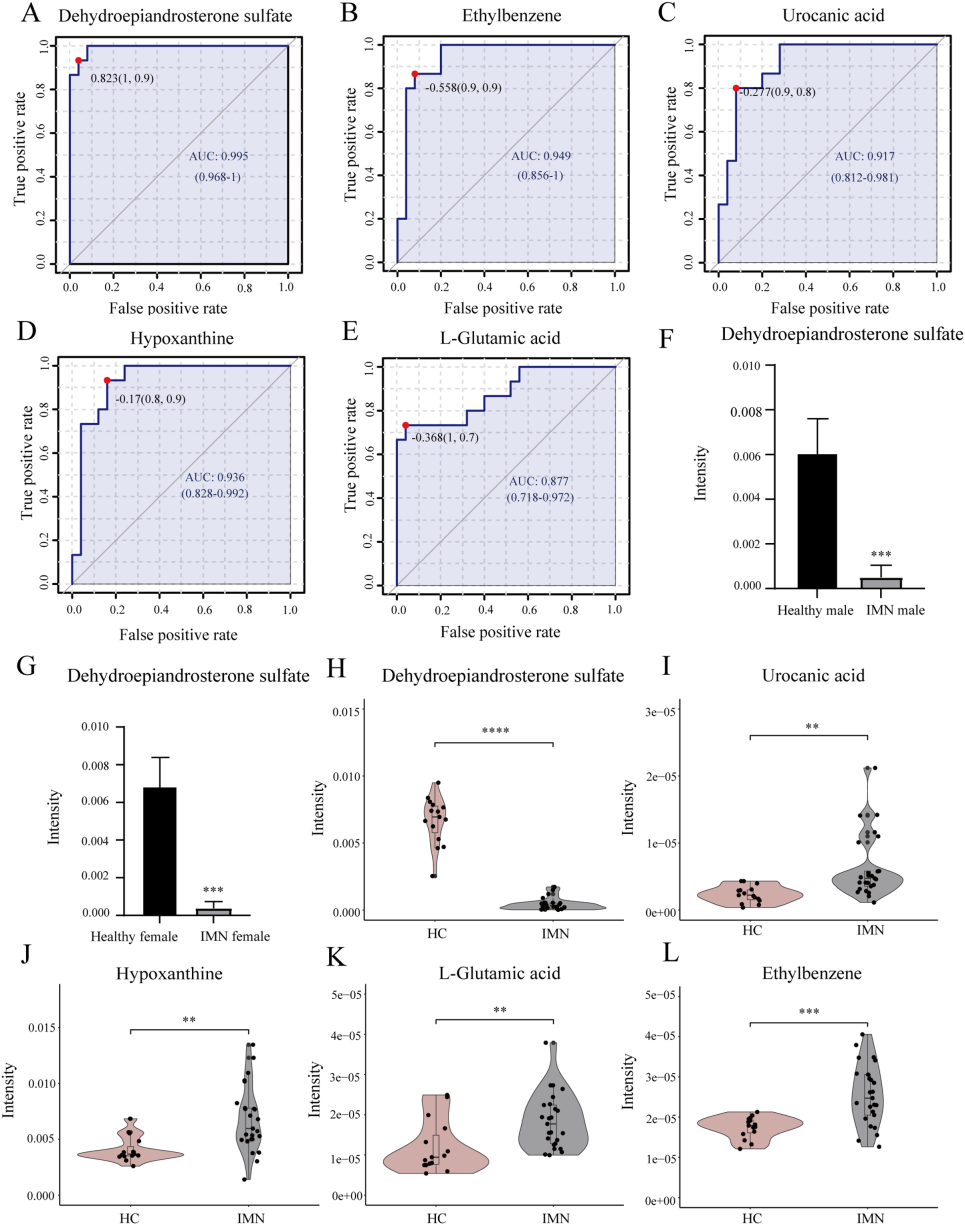
Biomarker analysis of key DEMs. (A) ROC of dehydroepiandrosterone sulfate with an AUC value of 0.995 (95% CI [0.968–1]). (B) ROC of ethylbenzene with a AUC value of 0.949 (95% CI [0.856–1]). (C) ROC of urocanic acid with an AUC value of 0.917 (95% CI [0.812–0.981]). (D) ROC of hypoxanthine with an AUC value of 0.936 (95% CI [0.828–0.992]). (E) ROC of L-glutamic acid with an AUC value of 0.877 (95% CI [0.718–0.972]). (F) The level of dehydroepiandrosterone sulfate in HC and IMN male groups. (G) The level of dehydroepiandrosterone sulfate in HC and IMN female groups. (H) The violin plot of the level of dehydroepiandrosterone sulfate in the HC and IMN groups. (I) The violin plot of the level of urocanic acid in the HC and IMN groups. (J) The violin plot of the level of hypoxanthine in the HC and IMN groups. (K) The violin plot of the level of L-glutamic acid in the HC and IMN groups. (L) The violin plot of the level of ethylbenzene in the HC and IMN groups. “**” means *p* < 0.01; “***” means *p* < 0.001; “****” means *p* < 0.0001.

In addition, we performed univariate ROC curve analysis for 215 differentially expressed metabolites using the biomarker analysis module of MetaboAnalyst 5.0. The area under the ROC curve (AUC) was used to identify the sensitivity and specificity of biomarkers, with AUC values greater than 0.7 indicating a good predictive model (Mandrekar, 2010; Răchieriu et al., 2021). Based on ROC curve analysis for individual biomarkers, the following five metabolites were brought to our attention with potential diagnostic significance, including dehydroepiandrosterone sulfate (AUC = 0.995, log2(FC) = −3.934), ethylbenzene (AUC = 0.949, log2(FC) = 0.551), hypoxanthine (AUC = 0.936, log2(FC) = 0.730), urocanic acid (AUC = 0.917, log2(FC) = 1.423), and L-glutamic acid (AUC = 0.877, log2(FC) = 0.616) (Figs. 4A–4E). Violin plots illustrated a statistically significant difference in these metabolites’ expression between in IMN group and the HC group (Figs. 4H–4L). In particular, DHEAS was significantly downregulated in IMN, since the studies showed that dehydroepiandrosterone sulfate was higher in males than in females (Rainey et al., 2002), we compared DHEAS for different genders separately between the IMN group and HC group. DHEAS was significantly down-regulated in membranous nephropathy in either men or women (*p* < 0.001) (Figs. 4F and 4G). In our study, we focused on histidine metabolism, steroid hormone biosynthesis, and purine metabolism pathways. L-glutamic acid, urocanic acid, imidazole-4-acetaldehyde, imidazoleacetic acid, and formiminoglutamic acid were enriched in the histidine metabolism pathway. Hypoxanthine, inosine, guanine, and guanosine were identified in the purine metabolism pathway (Table 2). It showed that the metabolites have a promising future as diagnostic markers for predicting diseases and will need more studies to confirm it (Bajaj et al., 2021; van der Velpen et al., 2019). Therefore, we inferred that serum metabolites were significantly altered in IMN patients, thus indicating alterations in metabolic pathways leading to some phenotypic changes, which have positive clinical implementation for exploring the pathogenic mechanisms and characteristics of idiopathic membranous nephropathy.

**Table 2:** The diagnostic performance of DEMs in significantly enriched KEGG pathways.

Metabolites	Super class	VIP	*p*-value	FC	Regulation	Pathway
L-Arginine	Organic acids and derivatives	1.34	0.018845352	1.28	Up	Arginine and proline metabolism
Guanidoacetic acid	Organic acids and derivatives	1.90	9.18E−05	0.63	Down	Arginine and proline metabolism
4-Guanidinobutanoic acid	Organic acids and derivatives	1.28	0.008772495	1.38	Up	Arginine and proline metabolism
4-Aminobutyraldehyde	Organic oxygen compounds	1.55	0.0002	1.60	Up	Arginine and proline metabolism
Formiminoglutamic	Organic acids and derivatives	2.07	0.0432	4.90	Up	Histidine metabolism
Urocanic acid	Organoheterocyclic compounds	1.66	0.0005	2.68	Up	Histidine metabolism
Imidazoleacetic acid	Organoheterocyclic compounds	1.75	5.32E−05	1.39	Up	Histidine metabolism
Imidazole-4-acetaldehyde	Organoheterocyclic compounds	2.26	7.91E−06	0.75	Down	Histidine metabolism
Uridine	Nucleosides, nucleotides, and analogues	1.52	0.0044	0.47	Down	Pyrimidine metabolism
Deoxycytidine	Nucleosides, nucleotides, and analogues	1.89	4.00E−06	0.61	Down	Pyrimidine metabolism
Pseudouridine	Nucleosides, nucleotides, and analogues	1.78	0.0007	1.65	Up	Pyrimidine metabolism
3-Aminoisobutanoic acid	Organic acids and derivatives	1.16	0.0389	1.28	Up	Pyrimidine metabolism
Pantothenic acid	Organooxygen compounds	1.74	0.0002	0.61	Down	Pantothenate and CoA biosynthesis
Dehydroepiandrosterone sulfate	Lipids and lipid-like molecules	2.66	8.56E−10	0.06	Down	Steroid hormone biosynthesis
Estrone glucuronide	Lipids and lipid-like molecules	1.90	0.0037	0.28	Down	Steroid hormone biosynthesis
Inosine	Nucleosides, nucleotides, and analogues	1.20	0.0385	1.70	Up	Purine metabolism
Hypoxanthine	Organoheterocyclic compounds	1.27	0.0002	1.66	Up	Purine metabolism
Guanine	Organoheterocyclic compounds	1.35	0.0141	0.44	Down	Purine metabolism
Guanosine	Nucleosides, nucleotides, and analogues	1.49	0.0105	0.43	Down	Purine metabolism
L-Glutamic acid	Organic acids and derivatives	1.39	0.0058	1.53	Up	Histidine metabolism, arginine and proline metabolism
Uracil	Organoheterocyclic compounds	1.21	0.0034	0.45	Down	Pyrimidine metabolism, pantothenate and CoA biosynthesis

**Note:**

DEMs, differentially expressed metabolites.

### Metabolite origin analysis

**Figure 5:**
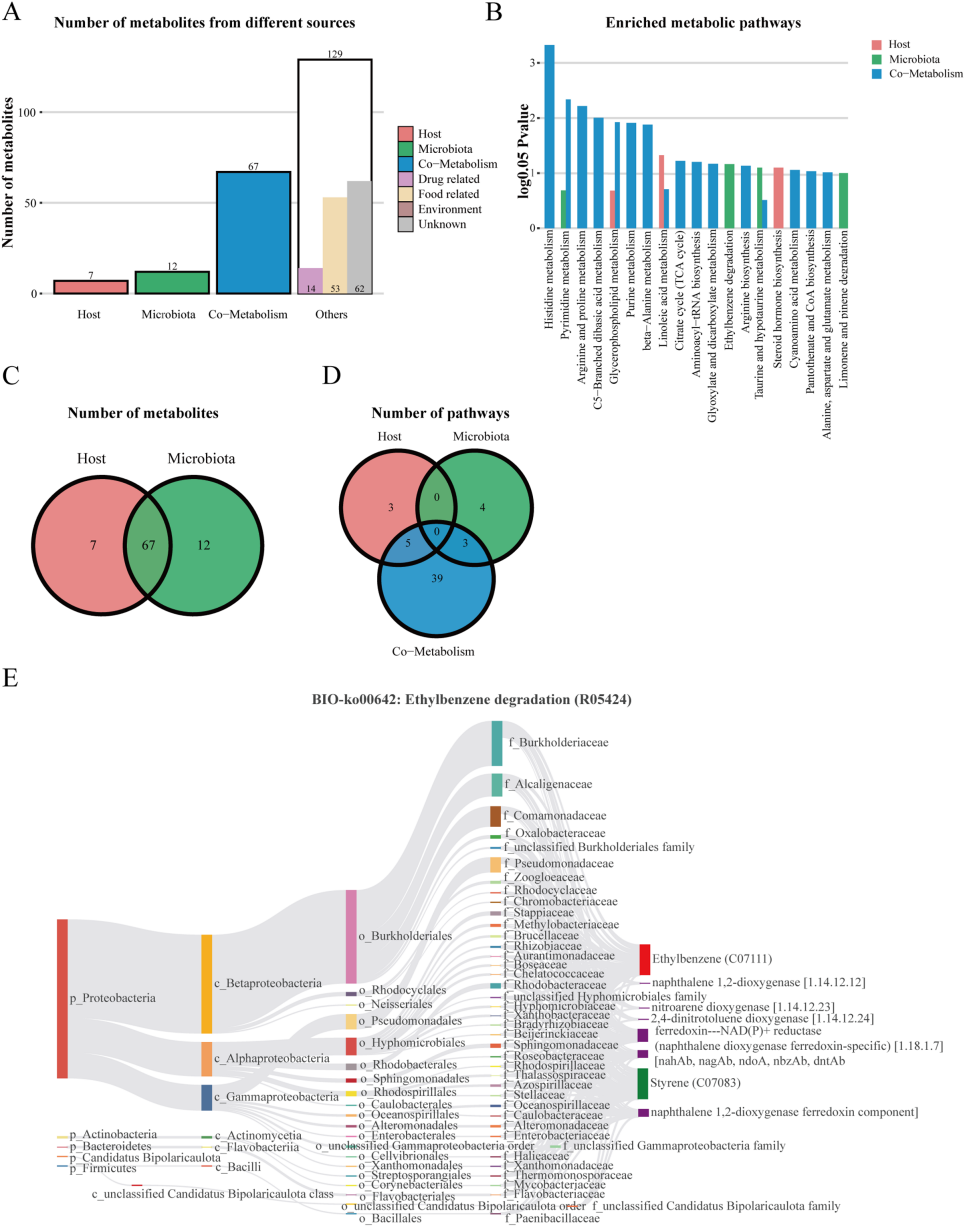
The host-microbiome origin analysis for DEMs. (A) The histogram of different source classifications of 215 DEMs. (B) The enriched metabolic pathways of DEMs in host-microbiome origin analysis. (C) Venn plot shows the overlaps of DEMs from the host and microbiota. (D) Venn plot shows the number of pathways in DEMs from the host, microbiota, and co-metabolism. (E) The biosankey network of the ethylbenzene degradation pathway for 215 DEMs (BIO-ko00642, R05424).

We performed host-microbiome origin analysis of differentially expressed metabolites using MetOrigin, aiming to effectively explore the interaction between gut microbes and serum metabolites. The sources of DEMs in the membrane nephropathy group and the control group could be classified as host, microbiota, co-metabolism, and other sources (food, drugs, environment, *etc*.). A total of seven DEMs were from the host only, 12 metabolites were from microbiota, 67 metabolites were from co-metabolism, and 129 metabolites were from others (Figs. 5A and 5C). Our study also performed metabolite pathway enrichment analysis (MPEA) of metabolic pathways for DEMs, which showed eight, seven and 47 metabolic pathways associated with the host, microbiota, and co-metabolism, respectively (Fig. 5D). Among the co-metabolized metabolic pathways, the DEMs were mainly involved in the histidine metabolism. The metabolites were mainly involved in the ethylbenzene degradation pathway for the microbiota (Fig. 5B). Spearman’s correlation analysis was used in metabolites and traceable gut microbes and presented in the Biosankey network. Therefore, we focused on histidine metabolism and ethylbenzene degradation pathways. For DEMs of co-metabolite, the 11 metabolic reactions were mainly contained in the histidine metabolism pathway (R00525, R01168, R02150, R02285, R02286, R02287, R02288, R02914, R03286, R04065, R10330). For example, in histidine metabolism (R01168), some DEMs were identified in this metabolic reaction, including histidine ammonia-lyase (4.3.1.3), L-histidine (C00135), and urocanic acid (C00785). The biosankey network revealed that the phylum proteobacteria, phylum actinobacteria, phylum bacteroidetes, phylum firmicutes, class gammaproteobacteria, class alphaproteobacteria, class actinomycetia, and class bacilli were closely related to these DEMs implicated in the histidine metabolism (R01168) (Fig. S1). Almost all of these metabolic reactions showed the same trend. In addition, in the microbiome, metabolites were mainly enriched in the BIO-ko00642 ethylbenzene degradation pathway, involving four metabolic reactions (R05424, R05425, R05440, R05745), with the main relevant metabolites being ethylbenzene (C07111), styrene (C07083), ferredoxin-NAD(P)+ reductase (naphthalene dioxygenase ferredoxin-specific) (1.18.1.7), and naphthalene 1,2-dioxygenase ferredoxin component. The metabolic reaction R05424 was shown in Fig. 5E. The results indicated that these differentially expressed metabolites were mainly from phylum proteobacteria, phylum actinobacteria, class beta proteobacteria, class alphaproteobacteria, class gammaproteobacteria, family burkholderiaceae, family alcaligenaceae, family comamonadaceae, and family pseudomonadaceae, with the same trend in the other three metabolic reactions (Fig. 5E). MetOrigin performed a functional analysis based on metabolites traceability, integrated correlation results of statistical and biological significance, and finally obtained biomarkers with high accuracy.

### Correlation network analysis

**Figure 6:**
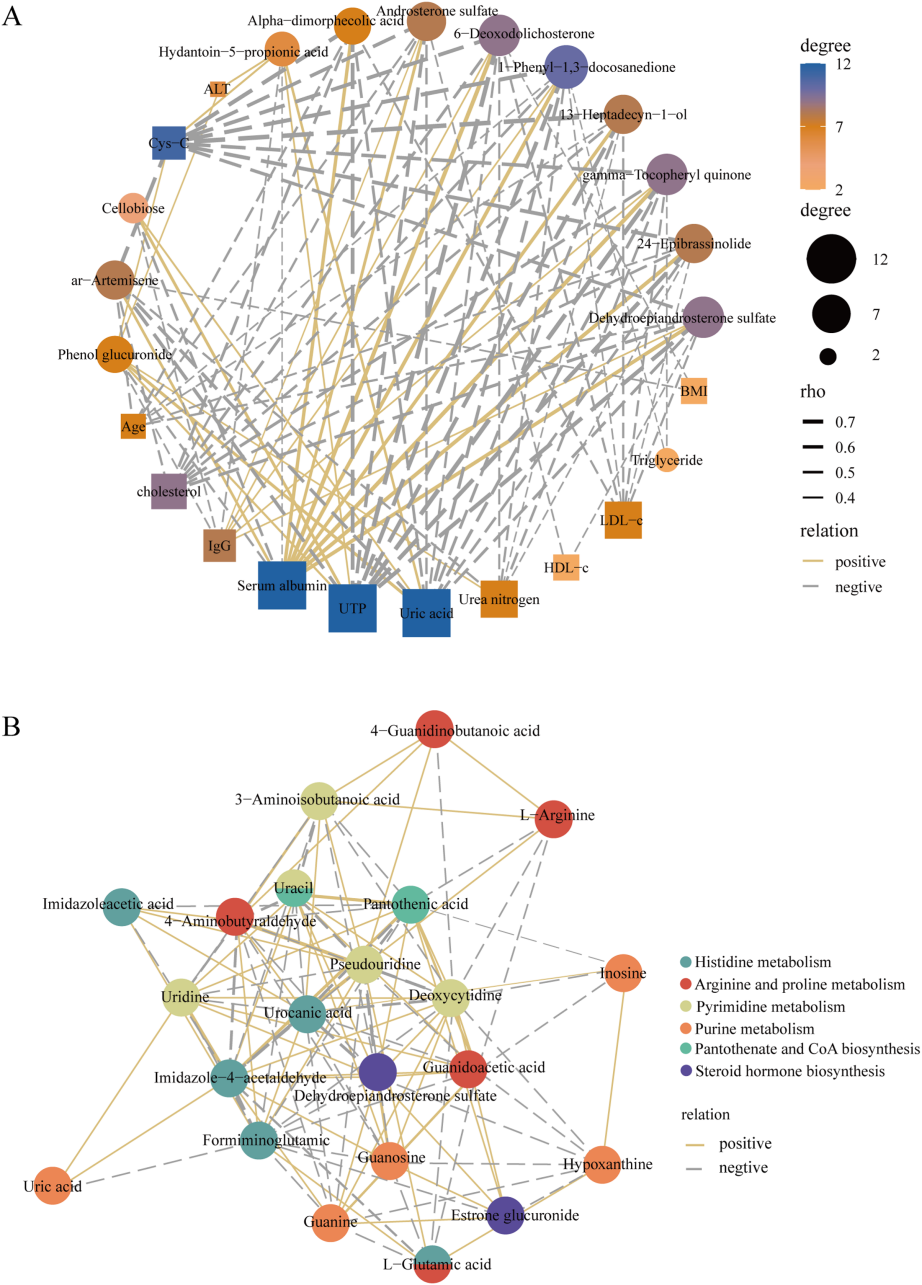
Construction of the correlation network. (A) The correlation network of key DEMs ( VIP > 1.5 and |log2 FC| > 3) and clinical indicators in the IMN and HC groups. (B) The correlation network of DEMs in significantly enriched KEGG pathways.

To better elucidate the interactions of differently expressed metabolites, we screened 12 significantly expressed metabolites under the criteria of VIP > 1.5 and |log2FC| > 3. The DEMs were subjected to Spearman correlation analysis with clinical indicators. The results showed that dehydroepiandrosterone sulfate (*r* = 0.63, *p* < 0.0001), 24-epibrassinolide (*r* = 0.64, *p* < 0.0001), gamma-tocopheryl quinone (*r* = 0.69, *p* < 0.0001), and androsterone sulfate (*r* = 0.53, *p* < 0.001) were positively correlated with the level of serum albumin. This positive correlation also existed in these metabolites with IgG. Among them, dehydroepiandrosterone sulfate was negatively correlated with LDL (*r* = −0.42, *p* < 0.01), UTP (*r* = −0.68, *p* < 0.0001), *i.e*., 24 h proteinuria, and cholesterol (*r* = −0.55, *p* < 0.001) (Fig. 6A). In addition, for KEGG pathway analysis, we selected six pathways with significant enrichment of DEMs and performed spearman correlation analysis in the metabolites identified on these pathways. We found that several key pathways were closely linked, including histidine metabolism, purine metabolism, arginine and proline metabolism, pantothenate and CoA biosynthesis, pyrimidine metabolism, and steroid hormone biosynthesis KEGG pathways (Fig. 6B). Therefore, there were metabolic disorders of amino acid, nucleotide, and steroid hormones in IMN patients.

## Discussion

As an organ-specific autoimmune disease, IMN results from combined factors such as immune abnormalities and genetic and environmental factors (van de Logt et al., 2019). Early diagnosis and treatment can delay or prevent the progression of IMN to ESRD. We revealed the serum metabolite profiles in IMN patients based on a non-targeted metabolomics approach and committed to finding a non-invasive diagnostic method. Our study identified 652 metabolites in whole serum samples, and 215 metabolites were defined as DEMs. Most of them were classified as lipids and lipid-like molecules and organic acids and derivatives. For the KEGG pathway analysis, these DEMs were mainly involved in histidine metabolism, pyrimidine metabolism, purine metabolism, arginine and proline metabolism, and steroid hormone biosynthesis. The result reflected that there were disorders of amino acids, nucleotides, and steroids hormones metabolism in the IMN group. Among them, we focused on the several DEMs significantly enriched in these pathways, such as dehydroepiandrosterone sulfate, hypoxanthine, urocanic acid, and L-glutamic acid. These metabolites were interlinked in the correlation analysis and considered reliable diagnostic markers in ROC curve analysis. It has been reported that disorders of purine metabolism were related to diseases such as gout and diabetic nephropathy (Liu et al., 2019; Xia et al., 2009; Zhao et al., 2005). Hypoxanthine was the substrate for xanthine oxidoreductase (XOR), which promoted the generation of reactive oxygen species (ROS) in tissues (Day et al., 2016). Accumulation of purine metabolites and oxidative stress led to tubulointerstitial and glomerular damage and impaired renal function (Dissanayake et al., 2022). Some studies revealed that there were high-level histidine and urocanic acid for patients with protein malnutrition (Bernard, Gallo & Krutmann, 2019). Urocanic acid and dehydroepiandrosterone sulfate were also thought to be related to autoimmune diseases (Bernard, Gallo & Krutmann, 2019; Kanda, Hoashi & Saeki, 2019).

We also performed a correlation analysis between several significantly expressed metabolites and clinical indicators. We found that dehydroepiandrosterone sulfate (DHEAS), gamma-tocopheryl quinone, and androsterone sulfate were positively related to serum albumin and IgG but negatively related to UTP, triglycerides, and LDL. Particularly, DHEAS has come to our attention that was considered the most reliable diagnostic marker in the biomarker analysis. In our study, DHEAS was expressed as a down-regulated DEM in IMN patients. It was more like a protective factor, which could aggravate the progression of membranous nephropathy in low serum levels. Dehydroepiandrosterone sulfate is an androgen precursor secreted by the zona reticularis of the adrenal gland and is the product of cholesterol metabolism bound to albumin in the serum (Baulieu & Robel, 1998; Dhatariya & Nair, 2003; Kroboth et al., 1999). Several studies showed that low serum DHEAS levels were linked to age-related diseases and immune system disorders, such as cardiovascular disease, diabetes mellitus, reduced immunocompetence, and systemic lupus erythematosus (SLE) (Dhatariya & Nair, 2003; Tchernof & Labrie, 2004). DHEAS deficiency may be related to poor prognosis in ESRD patients (Kakiya et al., 2012), and research on the pathogenic mechanisms of patients with membranous nephropathy is still lacking. Our study was based on the non-targeted metabolomics approach, and the results indicated that DHEAS was significantly down-regulated in the membranous nephropathy group. The results revealed comprehensive metabolic abnormalities in patients with membranous nephropathy. Combined with ROC curve analysis, DHEAS was considered the most reliable diagnostic marker (AUC = 0.995, log2(FC) = −3.93). It’s valuable for the clinical non-invasive diagnosis and the exploration of treatments for membranous nephropathy.

Although humoral immunity played a dominant role in the pathogenesis of IMN, some research suggested that the imbalance of the subsets of helper T (Th) cells may also be related to the pathogenic environment of idiopathic membranous nephropathy (Masutani et al., 2004; Zhao et al., 2021). Regulatory T (Treg) cells were important in the maintenance of immune tolerance by down-regulating the function of effector CD4(+) or CD8(+) T cells and secreting IL-10, IL-35, and TGF-β (Quiroga et al., 2012; Zhao et al., 2021). Fervenza et al. (2010) illustrated that the proportion of Treg cells in IMN serum was reduced, thereby undermining the immune tolerance and promoting antibodies’ autoimmune response. DHEA has been shown to induce Treg activity, promote Th1-type cytokines production and interfere with the synthesis of Th2-type cytokines (Kanda, Hoashi & Saeki, 2019). DHEA is the active form of DHEAS and can be converted into circulation (Rutkowski et al., 2014). In our study, DHEAS was identified as a significantly down-regulated DEM in the IMN group, which was significantly correlated with the level of several clinical indicators such as serum albumin, IgG, UTP, and cholesterol. The disturbance of steroid hormones metabolism reduced the level of DHEA and DHEAS, thus inducing the Treg cells’ activity, further leading to an imbalance in subsets of Th cells, creating an immune intolerant environment that promoted the autoimmune response to antibodies. Then, immune complexes were deposited on the interstitial space between the podocytes and the GBM and disrupted the glomerular filtration barrier, leading to the progression of IMN (Ronco et al., 2021; Ronco & Debiec, 2020).

Furthermore, we conducted traceability analysis for the identified DEMs to explore the interaction between metabolome and the traceable gut microbes. We found that seven DEMs were from the host only, 12 DEMs were from microbiota, 67 DEMs were from co-metabolism, and the DEMs were mainly enriched in the histidine metabolism for co-metabolized metabolic pathways and significantly enriched in ethylbenzene degradation pathway for the microbiota. The result revealed that phylum proteobacteria, phylum actinobacteria, and class gammaproteobacterial have a remarkable correlation with DEMs in these two pathways. The interaction between gut microbiota and the kidney, also known as the gut-kidney axis, has been emerging (Chen et al., 2019b; Giordano et al., 2021; Linh et al., 2022). Some studies indicated that dysbiosis of the gut microbiota could lead to an imbalance between the immune tolerance and immune response, resulting in the production of autoantibodies and inflammatory factors and leading to the progression of kidney disease (Bian et al., 2022; Chen et al., 2019b). Vaziri et al. (2013) revealed an increased abundance of actinobacteria, phylum proteobacteria, and class gammaproteobacterial in ESRD patients, which was consistent with our results. And these intestinal microbes were associated with several metabolites, such as histidine ammonia-lyase, urocanic acid, L-glutamic acid, ethylbenzene, and styrene in IMN patients. Therefore, we inferred that such disturbances of gut microbiota and metabolites were also present in membranous nephropathy, leading to the progression of kidney disease.

Our research still has some limitations, and we can further analyze the profiles of urinary metabolites in IMN patients simultaneously to make the results more feasible. In addition, it is also feasible to increase the sample size. Although still some way from replacing the invasive gold standard of renal puncture biopsy, using high-throughput metabolomics techniques for clinical diagnoses and treatment still holds promise and needs further exploration.

## Conclusion

Our study demonstrated that there were disorders of amino acids, nucleotides, and steroids hormones metabolism in IMN patients. The down-regulation of DHEAS may be related to the imbalance of the immune environment in IMN patients. In addition, the gut microbiota and metabolite disturbances were present in IMN patients for host-microbiome origin analysis.

## Supplemental Information

10.7717/peerj.15167/supp-1 Supplemental Information 1 Flowchart of the metabolomics analysis in serum samples from healthy control and IMN patients.

10.7717/peerj.15167/supp-2 Supplemental Information 2 Raw data. Clinical characteristics

10.7717/peerj.15167/supp-3 Supplemental Information 3 Differentially Expressed Metabolites.

10.7717/peerj.15167/supp-4 Supplemental Information 4 Metabolite origin analysis of DEMs. The biosankey network of histidine metabolism pathway for 215 DEMs (BIO-ko00340, R01168).

10.7717/peerj.15167/supp-5 Supplemental Information 5 Inspection process quality control. EIC diagram of internal standard positive ions in blank samples and QC samples. EIC: Extracted Ion Chromatogram.

10.7717/peerj.15167/supp-6 Supplemental Information 6 Inspection process quality control. EIC diagram of internal standard negative ions in blank samples and QC samples.

10.7717/peerj.15167/supp-7 Supplemental Information 7 Inspection process quality control. EIC diagram of internal standard positive ions in the QC samples.

10.7717/peerj.15167/supp-8 Supplemental Information 8 Inspection process quality control. EIC diagram of internal standard negative ions in the QC samples.

10.7717/peerj.15167/supp-9 Supplemental Information 9 Supplementary Materials and Methods.

## Abbreviations

DEMs: Differentially expressed metabolites
DHEAS: Dehydroepiandrosterone sulfate
IMN: Idiopathic membranous nephropathy
SMN: Secondary membranous nephropathy
GBM: Glomerular basement membrane
KEGG: Kyoto Encyclopedia of Genes and Genomes
ESRD: End-stage renal disease
PCA: Principal component analysis
OPLS-DA: Orthogonal projections to latent structures-discriminant analysis
AUC: Area under the ROC curve
ROC: Receiver operating characteristic curve
VIP: Variable importance in the projection
QC: Quality control

## Additional Information and Declarations

### Data Availability

The following information was supplied regarding data availability: The data is available at figshare: Ye, Mingjun; Tang, Donge; Li, Weilong; Ma, Chiyu; Zeng, Zhipeng; Liao, Shengyou; et al. (2023): MS1(POS+NEG+QC). figshare. Dataset. https://doi.org/10.6084/m9.figshare.21532893.v1.
