# Supplementary figures and images for: Serum metabolomics analysis reveals metabolite profile and key biomarkers of idiopathic membranous nephropathy
Source: PeerJ. 2023 Apr 6;11:e15167. doi: 10.7717/peerj.15167 (PMC10083006; doi:10.7717/peerj.15167)

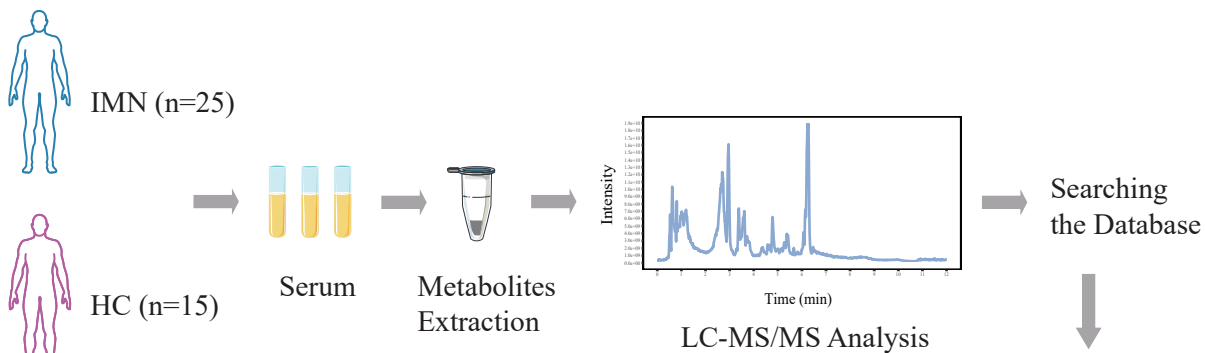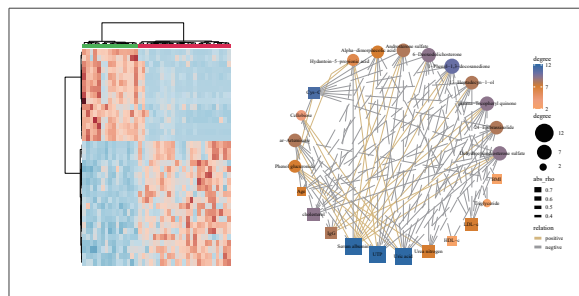

Bioinformatics Analysis

Supplement: Supplemental Information 1 [file peerj-11-15167-s001.pdf]

BIO-ko00340: Histidine metabolism (R01168)

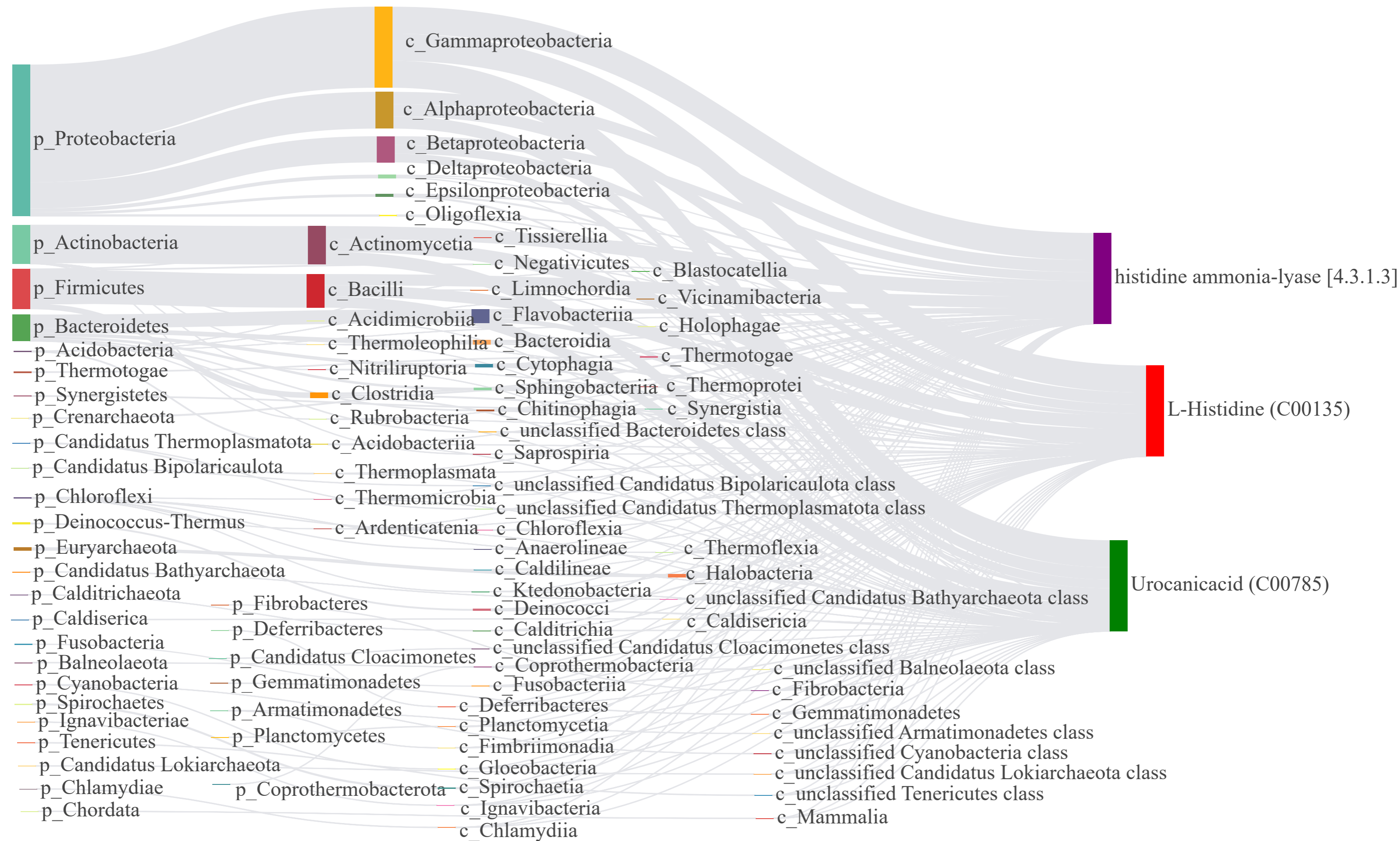

Supplement: Supplemental Information 4 — The biosankey network of histidine metabolism pathway for 215 DEMs (BIO-ko00340, R01168). [file peerj-11-15167-s004.pdf]

1\_QC01 1\_QC02 1\_QC03 1\_QC04 1\_QC05  
1\_QC06 1\_QC07 1\_QC08 1\_QC09 2\_Blank1  
2\_Blank2 2\_Blank3 2\_Blank4

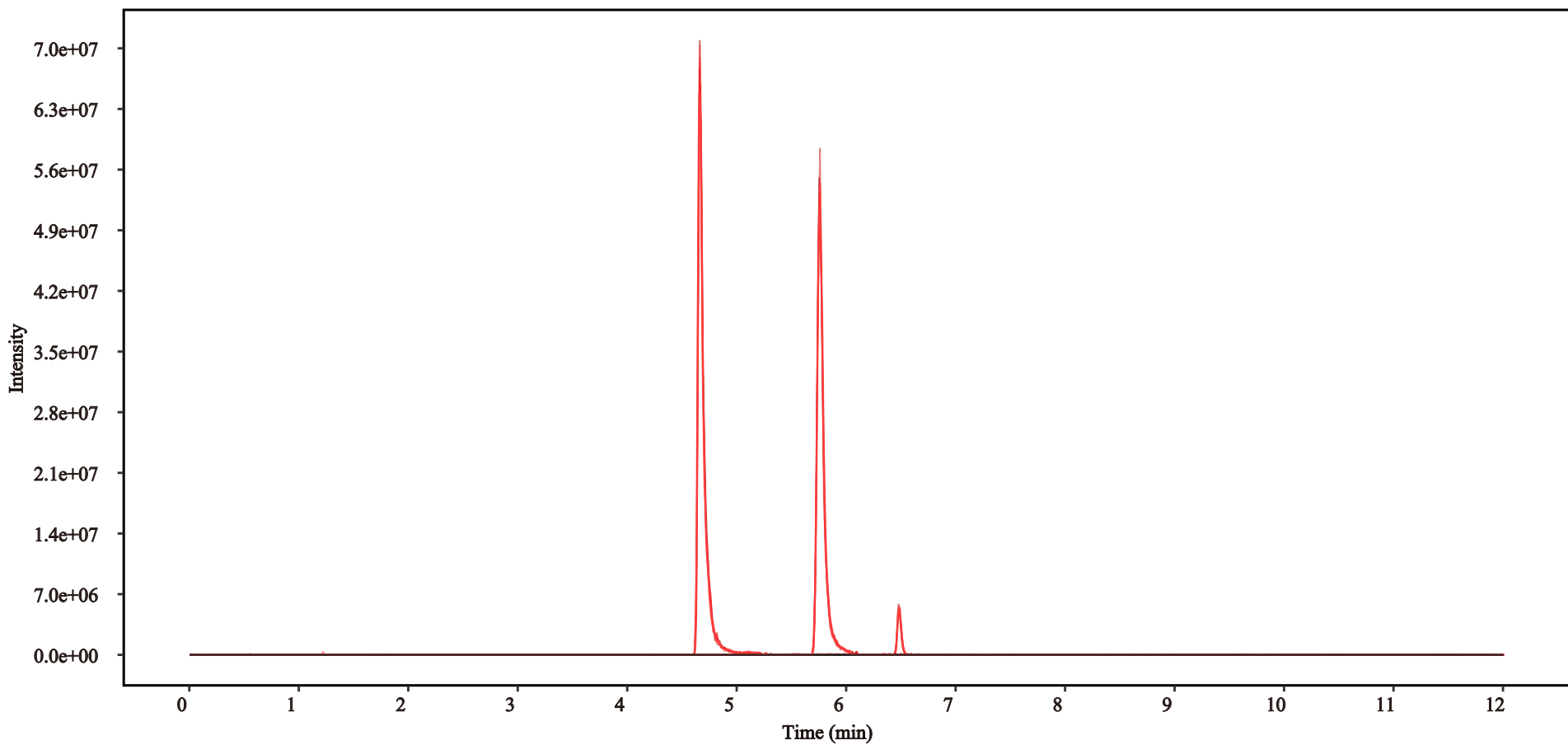

Supplement: Supplemental Information 5 — EIC diagram of internal standard positive ions in blank samples and QC samples. EIC: Extracted Ion Chromatogram. [file peerj-11-15167-s005.pdf]

1\_QC01 1\_QC02 1\_QC03 1\_QC04 1\_QC05  
1\_QC06 1\_QC07 1\_QC08 1\_QC09 2\_Blank1  
2\_Blank2 2\_Blank3 2\_Blank4

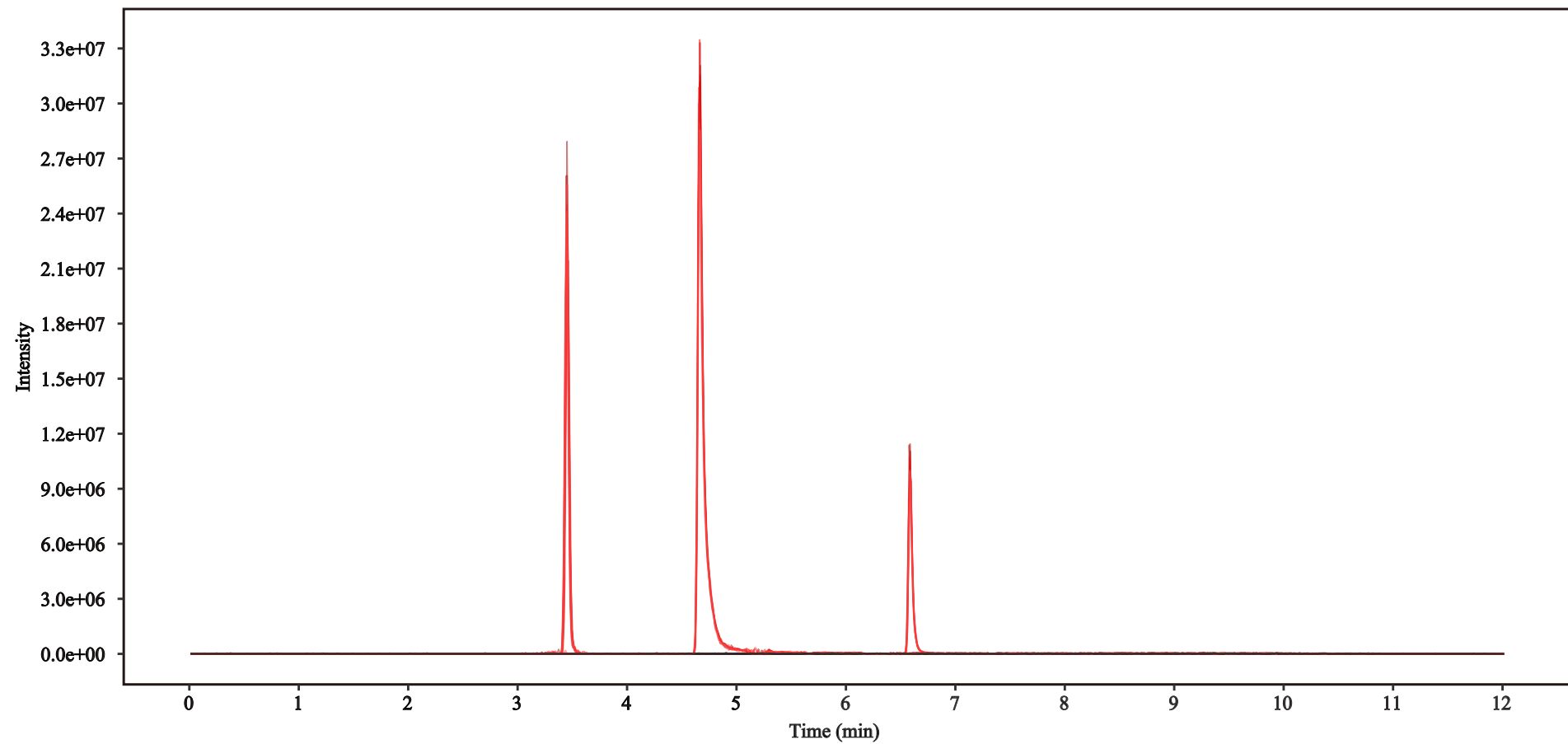

Supplement: Supplemental Information 6 — EIC diagram of internal standard negative ions in blank samples and QC samples. [file peerj-11-15167-s006.pdf]

1\_QC01 1\_QC02 1\_QC03 1\_QC04 1\_QC05  
1\_QC06 1\_QC07 1\_QC08 1\_QC09

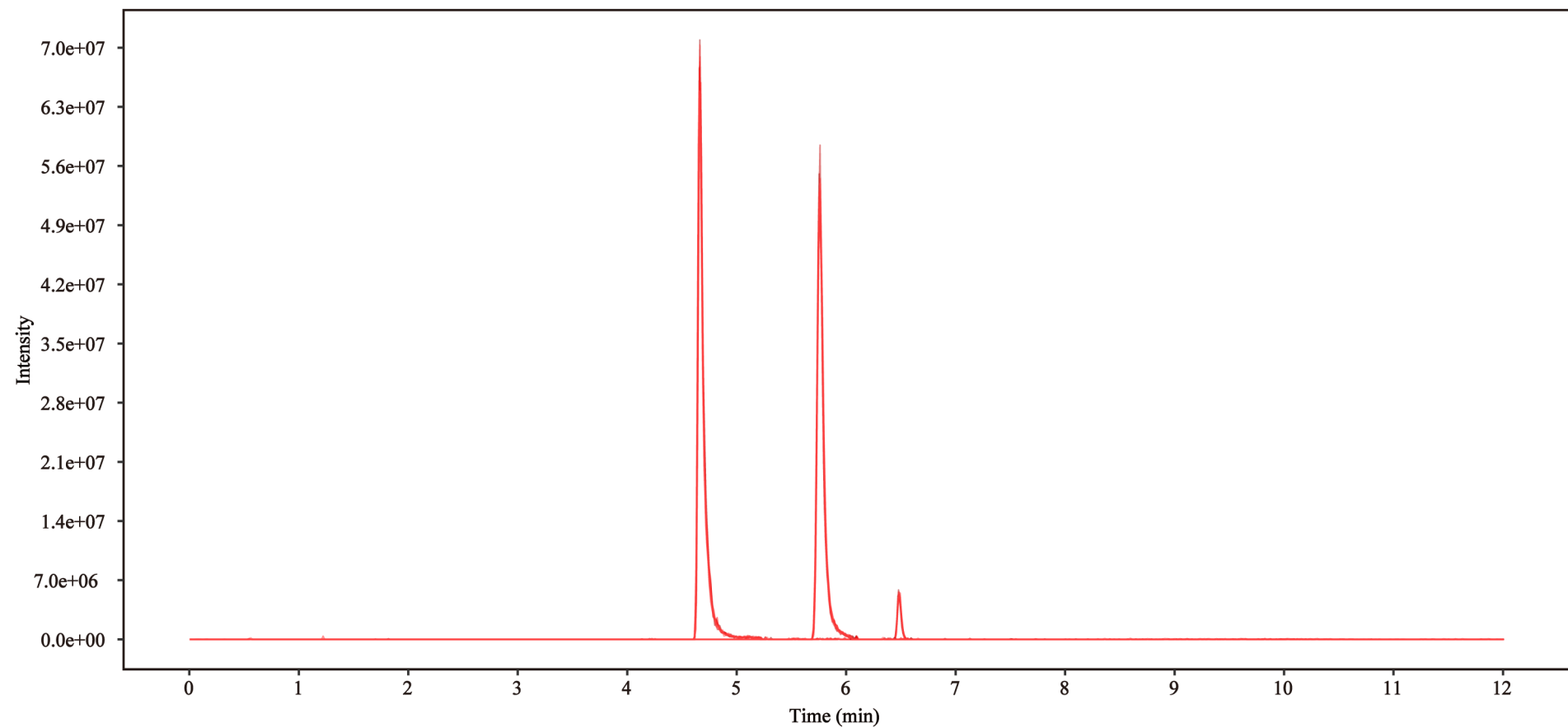

Supplement: Supplemental Information 7 — EIC diagram of internal standard positive ions in the QC samples. [file peerj-11-15167-s007.pdf]

1\_QC01 1\_QC02 1\_QC03 1\_QC04 1\_QC05  
1\_QC06 1\_QC07 1\_QC08 1\_QC09

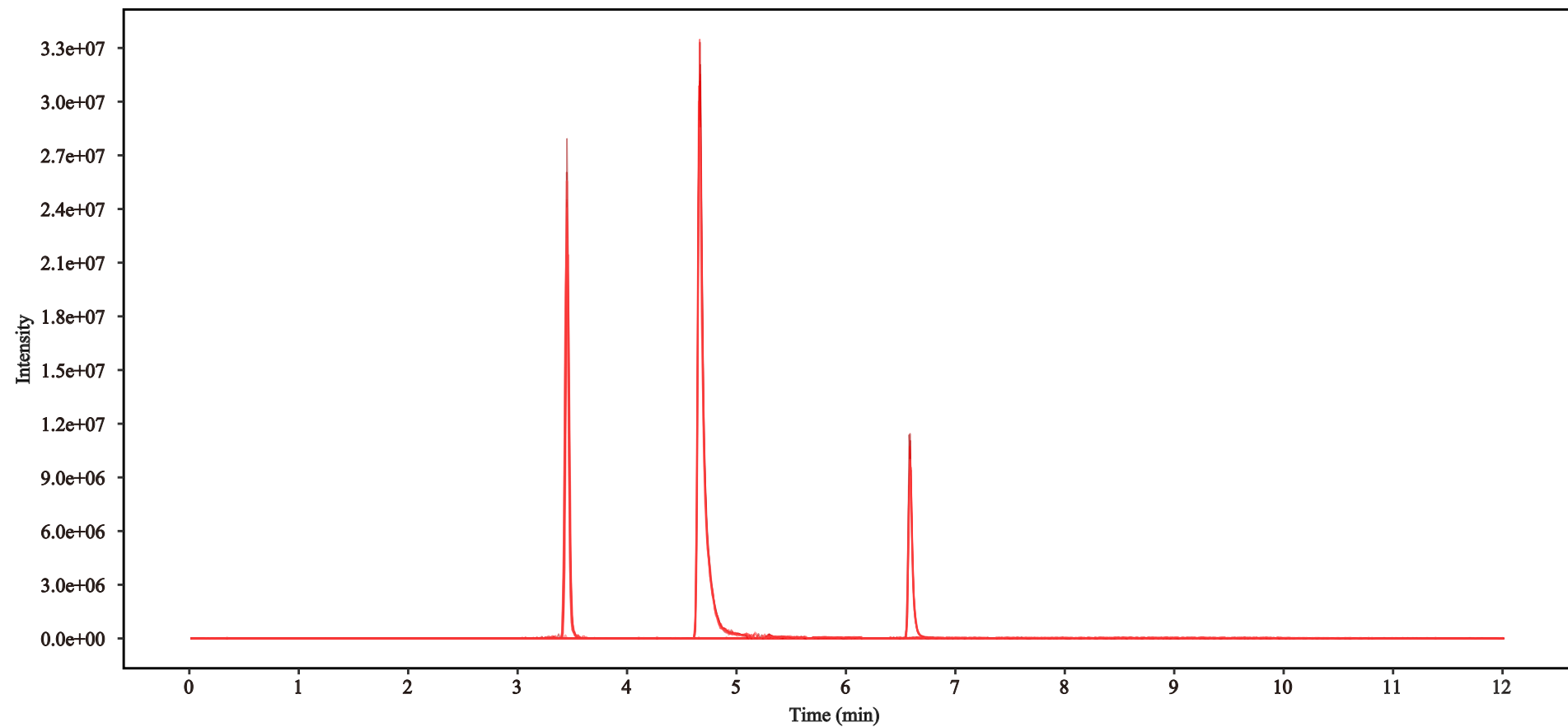

Supplement: Supplemental Information 8 — EIC diagram of internal standard negative ions in the QC samples. [file peerj-11-15167-s008.pdf]
