# Supplementary material for: Serum metabolomics analysis reveals metabolite profile and key biomarkers of idiopathic membranous nephropathy
Source: PeerJ. 2023 Apr 6;11:e15167. doi: 10.7717/peerj.15167 (PMC10083006; doi:10.7717/peerj.15167)
Supplement: Supplemental Information 9 [file peerj-11-15167-s009.pdf]

Supplementary materials

2 Materials and Methods

2.1 Patient Information

A total of 25 patients diagnosed with idiopathic membranous nephropathy were recruited from the First Affiliated Hospital of Jinan University. The ages of all IMN patients ranged from 40 to 73 years. Likewise, we also recruited 15 age-matched people for healthy controls. All IMN patients underwent renal biopsy to confirms the diagnosis. The inclusion criteria of our study were as follows: all patients were initial diagnosed with idiopathic membranous nephropathy by renal biopsy in the observation group; there were no history of kidney surgery or taking drugs for kidney damage prior to participating in this study; no use of hormones or immunosuppressants. As for the exclusion criteria, we eliminated the patients with serious organ dysfunction such as heart, liver, kidney or lung, and patients with Hepatitis B associated nephritis lupus nephritis, tumor-associated membranous nephropathy, organ transplantation, syphilis antibody positive, and heavy metal exposure. Particularly, patients with poor compliance were also excluded in our research. All participants are Chinese and have the same dietary habits and lifestyle. Our study was approved by the ethics committee of the First Affiliated Hospital of Jinan University (KY-2021-018). We collected the serum samples after obtaining the written informed consent of all participants.

2.2 Metabolomics analysis

2.2.1 Metabolites Extraction

100 μL of sample was transferred to an EP tube. After the addition of 400 μL of extract solution (acetonitrile: methanol = 1:1, containing isotopically-labelled internal standard mixture), the samples were vortexed for 30 s, sonicated for 10 min in ice-water bath, and incubated for 1 h at -40 °C to precipitate proteins. Then the sample was centrifuged at 12000 rpm for 15 min at 4 °C. 120 μL of resulting supernatant was transferred to a sample vial to prepare for further LC-MS/MS analysis. The quality control (QC) sample was prepared by mixing an equal aliquot of the supernatants from all of the samples.

2.2.2 LC-MS/MS Analysis

LC-MS/MS analyses were performed using an UHPLC system (Vanquish, Thermo

Fisher Scientific) with an ACQUITY UPLC BEH Amide column (2.1 mm × 100 mm, 1.7 μm, Waters) coupled to Q Exactive HFX mass spectrometer (Orbitrap MS, Thermo). The mobile phase A consisted of 25 mmol/L ammonium acetate and 25 mmol/L ammonia hydroxide in water (pH = 9.75), and the mobile phase B was 100% acetonitrile. The auto-sampler temperature was maintained at 4°C, and the column temperature was set at 30°C. The injection volume was set at 3 μL, and the flow rate was set at 0.5 mL/min. The following elution gradient was applied: 0-0.5min, 95% B; 0.5-7min, 95%-65%B; 7-8min, 65%B-40%B; 8-9min, 40%B; 9-9.1min, 40%-95%B; 9.1-12min, 95%B. The QE HFX mass spectrometer was used for its ability to acquire MS/MS spectra on information-dependent acquisition (IDA) mode in the control of the acquisition software (Xcalibur, Thermo). In this mode, the acquisition software continuously evaluates the full scan MS spectrum. The ESI source conditions were set as following: sheath gas flow rate as 30 Arb, Aux gas flow rate as 25 Arb, capillary temperature 350 °C, full MS resolution as 60000, MS/MS resolution as 7500, collision energy as 10/30/60 in NCE mode, spray Voltage as 3.6 kV (positive) or -3.2 kV (negative), respectively.

2.2.3 Data preprocessing and annotation

The raw data were converted to the mzXML format using ProteoWizard and processed with an in-house program, which was developed using R software version 4.0.4 and based on XCMS software version 3.8.2 by Biotree Biotech Co. (Shanghai, China), for peak detection, extraction, alignment, and integration(Smith et al. 2006). The parameters in XCMS were set as follows: central wave setting for feature detection (Δm/z = 10 ppm, peak width = c (5, 20)); obiwarp setting for retention time correction (profStep = 0.1); parameters including minfrac = 0.5, bw = 5 and mzwid = 0.015 for chromatogram comparison. The data matrices containing the sample names, retention times, m/z pairs, and normalized peak intensities were obtained. The metabolites were identified by matching with the m/z, retention time, and the MS/MS fragmentation information in the Human Metabolome Database (HMDB, https://hmdb.ca/), MassBank of North America (MoNA, https://mona.fiehnlab.ucdavis.edu/) database, Metlin database (https://metlin.scripps.edu/) and authentic standards introduced to the

in-house MS2 database established by Biotree Biotech Co. (Shanghai, China). The cutoff of MS2 score for annotation was set at 0.3. An accuracy error of 10 ppm was set for MS searching.

2.2.4 Bioinformatics methods

In this study, the missing values were filled up by half of the minimum value. Also, internal standard normalization method was employed in this data analysis. The final dataset containing the information on peak number, sample name, retention times, m/z pairs, and normalized peak area was imported to SIMCA16.0.2 software package (Sartorius Stedim Data Analytics AB, Umea, Sweden) for multivariate analysis. Data was scaled and logarithmic transformed to minimize the impact of both noise and high variance of the variables. After these transformations, principle component analysis (PCA), an unsupervised analysis that reduces the dimension of the data, was carried out to visualize the distribution and the grouping of the samples. 95% confidence interval in the PCA score plot was used as the threshold to identify potential outliers in the dataset. In order to visualize group separation and find significantly changed metabolites, supervised orthogonal projections to latent structures-discriminate analysis (OPLS-DA) was applied. Then, a 7-fold cross validation was performed to calculate the value of R2 and Q2. R2 indicates how well the variation of a variable is explained and Q2 means how well a variable could be predicted. To check the robustness and predictive ability of the OPLS-DA model, a 200 times permutations was further conducted. Afterward, the R2 and Q2 intercept values were obtained. Here, the intercept value of Q2 represents the robustness of the model, the risk of overfitting and the reliability of the model, which will be the smaller the better. Furthermore, the value of variable importance in the projection (VIP) of the first principal component in OPLS-DA analysis was obtained. It summarizes the contribution of each variable to the model. *P*-values were derived from a two-tailed student's t-test on normalized peak areas of differentially expressed metabolites between the two groups. Fold change was derived from the univariate analysis in IMN cases versus controls. The metabolites with VIP>1 and p <0.05 (Student's t-tests) were considered as significantly changed metabolites. Furthermore, we used R version 4.0.4 to create the visual graphs as

described below. The permutation test of the OPLS-DA model was applied by the R package “ggplot2”, which was important in avoiding overfitting of the test model and in assessing the statistical significance of the model. The process quality control with good stability was shown in supplementary materials (Figure S2-5). Volcano plot and violin plots were performed with the R package “ggplot2”, and the radar chart was performed with R package “fmsb”. The pathway enrichment, biomarker analysis, and statistical analysis were visualized with MetaboAnalyst 5.0. Moreover, we used MetaboAnalyst 5.0 to create the heat map and receiver operating characteristic (ROC) curve analyses using the biomarker analysis module. Metorigin 2.0 was conducted on host-microbiome origin analysis of differentially expressed metabolites.

2.3 Statistical analysis

Our study performed the statistical analysis by GraphPad Prism 9.0 software, using two-tailed Student’s t-tests for measurement data. A *p*-value less than 0.05 was considered statistically significant. We applied Spearman correlation analysis to analyze correlations between DEMs and clinical parameters. The correlation analysis and correlation network were visualized with the OmicStudio tools.

Reference:

Smith CA, Want EJ, O'Maille G, Abagyan R, and Siuzdak G. 2006. XCMS: processing mass spectrometry data for metabolite profiling using nonlinear peak alignment, matching, and identification. *Anal Chem* 78:779-787. 10.1021/ac051437y
